# Osteopontin mediates survival, proliferation and migration of neural stem cells through the chemokine receptor CXCR4

**DOI:** 10.1186/s13287-015-0098-x

**Published:** 2015-05-22

**Authors:** Monika Rabenstein, Joerg Hucklenbroich, Antje Willuweit, Anne Ladwig, Gereon Rudolf Fink, Michael Schroeter, Karl-Josef Langen, Maria Adele Rueger

**Affiliations:** Department of Neurology, University Hospital of Cologne, Cologne, Germany; Cognitive Neuroscience, Institute of Neuroscience and Medicine (INM-3), Research Centre Juelich, Leo-Brandt-Straße, 52425 Juelich, Germany; Medical Imaging Physics, Institute of Neuroscience and Medicine (INM-4), Research Centre Juelich, Juelich, Germany

## Abstract

**Introduction:**

Osteopontin (OPN) is a phosphoglycoprotein with important roles in tissue homeostasis, wound healing, immune regulation, and stress responses. It is expressed constitutively in the brain and upregulated during neuroinflammatory responses; for example, after focal cerebral ischemia. To date, its effects on neural stem cells (NSC) remain to be elucidated and are, accordingly, the subject of this study.

**Method:**

Primary fetal rat NSC were cultured as homogenous monolayers and treated with different concentrations of OPN. Fundamental properties of NSC were assessed following OPN exposure, including proliferative activity, survival under oxidative stress, migration, and differentiation potential. To elucidate a putative action of OPN via the CXC chemokine receptor type 4 (CXCR4), the latter was blocked with AMD3100. To investigate effects of OPN on endogenous NSC in vivo, recombinant OPN was injected into the brain of healthy adult rats as well as rats subjected to focal cerebral ischemia. Effects of OPN on NSC proliferation and neurogenesis in the subventricular zone were studied immunohistochemically.

**Results:**

OPN dose-dependently increased the number of NSC in vitro. As hypothesized, this effect was mediated through CXCR4. The increase in NSC number was due to both enhanced cell proliferation and increased survival, and was confirmed in vivo. Additionally, OPN dose-dependently stimulated the migration of NSC via CXCR4. Moreover, in the presence of OPN, differentiation of NSC led to a significant increase in neurogenesis both in vitro as well as in vivo after cerebral ischemia.

**Conclusion:**

Data show positive effects of OPN on survival, proliferation, migration, and neuronal differentiation of NSC. At least in part these effects were mediated via CXCR4. Results suggest that OPN is a promising substance for the targeted activation of NSC in future experimental therapies for neurological disorders such as stroke.

**Electronic supplementary material:**

The online version of this article (doi:10.1186/s13287-015-0098-x) contains supplementary material, which is available to authorized users.

## Introduction

Osteopontin (OPN) is an acidic phosphoglycoprotein containing the adhesive motif arginine-glycine-aspartate that interacts with various cell-surface integrins such as αvβ1, αvβ3, and α5β1 (reviewed in [1]). In response to injury and inflammation, OPN expression is induced in a number of cells including macrophages, activated T cells, osteoclasts, fibroblasts, epithelial cells, and vascular smooth muscle cells [2–6]. Both a soluble isoform and an intracellular isoform exist (reviewed in [1]). OPN plays an important role in tissue homeostasis, wound healing, immune regulation, and stress response [7, 8]. Intriguingly, under inflammatory conditions, it can stimulate both pro- and anti-inflammatory processes, depending on concomitant circumstances [7, 9–13]. In the brain, OPN is expressed constitutively and is upregulated with neuroinflammation (that is, in the subacute stage of cerebral ischemia) 3–6 days after stroke [14–17]. In cerebral ischemia, OPN mediates neuroprotection via two distinct mechanisms: a direct neuroprotective effect on (cortical) neurons [18, 19], and an indirect neuroprotective effect of OPN mediated via the transcriptional regulation of inducible nitric oxide synthase and consecutive reduction of secondary tissue damage [20].

Endogenous neural stem cells (NSC) residing in the subventricular zone (SVZ) and in the hippocampal dentate gyrus of the adult mammalian brain are mobilized following cerebral ischemia [21–23]. NSC mediate regeneration and functional recovery after stroke by way of neurogenesis and replacement of lost neurons, but also via pleiotropic functions including neuroprotection, reduction of neuroinflammation, revascularization, and induction of plasticity (reviewed in [24]). Their ability to migrate to a site of injury, to survive in the local environment, and potentially form new neurons, is crucial for their function but is often impaired after stroke [25, 26], contributing to the insufficient capacity of the brain for self-repair and functional recovery. Thus, supporting the proliferation, survival, and migration of endogenous NSC seems to be a promising therapeutic approach in stroke [22, 23, 27–31].

OPN is a potent chemoattractant, promoting the migration of cells of monocyte/macrophage lineage [32] as well as of osteoclast precursors [33], mesenchymal stem cells [34], and hematopoietic stem cells [35]. In the brain, NSC are typically recruited to sites of brain injury by the cytokine stromal cell-derived factor (SDF)-1α that is expressed in the damaged tissue, acting on the CXC chemokine receptor type 4 (CXCR4). CXCR4 is expressed on various types of stem cells, including hematopoietic stem cells [36] and NSC [37]. Zhang et al. demonstrated that in cell lines from hepatocellular carcinoma, as an analogue of SDF-1, OPN can also bind to CXCR4 and promote cell migration [38].

To date, little is known about the effects of OPN on NSC. For neural progenitor cells grown in neurosphere cultures, OPN was suggested to increase proliferation [39] and migration [40, 41] by yet unknown mechanisms. We hypothesized that OPN promotes proliferation and migration of NSC through CXCR4 in vitro as well as in vivo as a potential means of mobilizing and attracting NSC to the brain after focal cerebral ischemia. Moreover, we examined the effects of OPN on the differentiation potential of NSC.

## Material and methods

### Cell culture

Primary NSC were cultured from fetal rat cortices at embryonic day 13.5 as serum-free monolayers [22]. Briefly, cells were plated on dishes coated with poly-L-ornithine and fibronectin, and expanded in Dulbeccos’s modified Eagle’s/F12 medium (Life Technologies, Darmstadt, Germany) plus N2 supplement (Gibco, Karlsruhe, Germany), penicillin/streptomycin, L-glutamine, and sodium pyruvate. As a mitogen, fibroblast growth factor (FGF)2 was included at 10 ng/ml throughout the experiments (Invitrogen, Karlsruhe, Germany). After first passaging, homogenous NSC cultures were re-plated at 10,000 cells per cm^2^. Only NSC from the second until the fifth passage were used for all experiments, in order to utilize unaltered primary cells.

### Immunocytochemistry

All immunocytochemical experiments were performed in triplicate.

#### Characterization of NSC

Typical characteristics of NSC in culture were confirmed immunocytochemically to verify homogeneity of the cultures. Cells were fixed with 4 % paraformaldehyde (PFA) and stained for: i) SRY (sex determining region Y) box 2 (SOX2) as a marker for undifferentiated (stem) cells (mouse monoclonal, dilution 1:1000, cat-# MAB2018, R&D Systems, Minneapolis, USA), and ii) their expression of the cytokine receptor CXCR4 (polyclonal rabbit, dilution 1:250, cat-# 2042, Abcam, Bristol, UK). To detect a potential effect of OPN on the expression of CXCR4, NSC were treated in the presence or absence of 3.125 μg/ml OPN for 24 h, then fixed in 4 % PFA and stained for CXCR4. For visualization of SOX2 and CXCR4, fluorescein-labeled anti-rabbit immunoglobulin (Ig)G were used (dilution 1:200, Invitrogen); all cells were additionally counterstained with Hoechst 33342 (Life Technologies).

#### Cell number assessment

To assess the effects of OPN on NSC numbers, recombinant rat osteopontin (OPN; R&D Systems) with concentrations ranging from 1.25–12.5 μg/ml was added to the cultures 1 h after re-plating. To assess the role of CXCR4 in mediating any effects of OPN on cell numbers, we blocked this receptor with the CXCR4-antagonist AMD3100 (Tocris Bioscience, Bristol, UK) at a concentration of 10 μM in some wells directly after re-plating, resulting in a pre-incubation time of NSC with AMD3100 of 1 h before adding OPN. After 72 h, dead cells were stained with propidium iodide (Life Technologies). All cells, irrespective of viability, were counterstained with Hoechst 33342 (Life Technologies) and representative pictures were taken using an inverted fluorescence phase-contrast microscope (Keyence BZ-9000E; Keyence, Neu-Isenburg, Germany). Ten images were taken per well of a 24-well plate, and both Hoechst-stained and propidium iodide-stained NSC were counted manually. To calculate the ratio of living cells, the number of propidium iodide-negative cells was divided by the total (Hoechst-stained) number of NSC in each sample, and mean values were established among equally treated samples. Results were expressed as percent of the control ± standard error of the mean (SEM).

#### Ki67 stainings

To directly assess the effects of OPN on the proliferation of NSC, cells were treated with OPN at a concentration of 6.25 μg/ml for 72 h starting at re-plating. Cells were fixed with 4 % PFA and were stained for the expression of the proliferation marker Ki67 (rabbit polyclonal, dilution of 1:500, cat-# ab15580, Abcam). For visualization, fluorescein-labeled anti-mouse IgG was used; all cells were additionally counterstained with Hoechst 33342. Representative pictures were taken using an inverted fluorescence phase-contrast microscope (Keyence BZ-9000E), and Ki67-positive, proliferating NSC were counted manually. To calculate the ratio of proliferating cells, the number of Ki67-positive cells was divided by the total cell number in each sample, and mean values were established among equally treated samples. Results were expressed as percent of the control ± SEM.

#### Oxidative stress assay

To assess the effects of OPN on NSC under oxidative stress, NSC were allowed to grow for 24 h after re-plating, and were then treated with 300 nM 30 % H_2_O_2_ for 24 h (Merck, Darmstadt, Germany). OPN at 6.25 μg/ml was added to NSC cultures either with plating (resulting in a pre-incubation time of 24 h before oxidative stress, pre-treatment), or at the same time as the addition of H_2_O_2_ (simultaneous treatment). After 24 h under oxidative stress with H_2_O_2_, NSC were co-stained with propidium iodide to label dead cells, and Hoechst 33342 to label all cells regardless of viability. Ten representative images were taken per well of the 24-well plate, and cells were counted manually. To calculate the ratio of dead cells, the number of propidium iodide-positive cells was divided by the total cell number in each sample, and mean values ± SEM were established among equally treated samples.

#### Low-density plating

To further assess the effects of OPN in another cell stress model, NSC were plated at very low density (266 cells/cm^2^) and grown in the presence or absence of OPN at 6.25 μg/ml. After 8 days, NSC were co-stained with propidium iodide to label dead cells, and Hoechst 33342 to label all cells regardless of viability. Five representative images were taken per well of the 24-well plate, and cells were counted manually. To calculate the ratio of living cells, the number of propidium iodide-negative cells was divided by the total cell number in each sample, and mean values ± SEM were established among equally treated samples.

#### NSC migration

NSC migration was analyzed via a trans-well migration assay using a modified Boyden chamber (CytoSelect™ 24-Well Cell Migration Assay, 8 μM pore size; Cell Biolabs, Inc., San Diego, USA) according to the manufacturer’s protocol. Briefly, NSC were seeded in the inserted upper chamber. To assess the role of CXCR4 in mediating effects of OPN on migration, we blocked the receptor with AMD3100 at a concentration of 10 μM in half of the wells directly after plating for 1 h as pre-incubation before adding OPN. After 1 h, either 10 μg/ml OPN, 10 % fetal calf serum (FCS) as a positive control, or phosphate-buffered saline as a negative control, were added to the lower chambers of the modified Boyden chamber. After 48 h, the non-migrating cells on the upper side of the inserts were removed with a cotton tip. The migrating cells on the lower side of the inserts were stained with Cell Stain Solution for 10 minutes (Cell Biolabs, Inc.). Subsequently, they were washed with phosphate-buffered saline and allowed to air dry, before they were incubated with the Extraction Solution (Cell Biolabs, Inc.). The optical density of each sample was measured at 560 nm in a plate reader (FLUOstar Omega; BMG LABTECH, Ortenberg, Germany). Mean values ± SEM were established among equally treated samples, and results were expressed as percent of the control.

#### NSC differentiation

The differentiation potential of OPN-treated cells was investigated after withdrawal of the mitogen FGF2 during the expansion phase. After 7 days, the culture medium was replaced by Neurabasal Medium with GlutaMAX, penicillin/streptomycin, L-glutamine, sodium selenite, B27, NT3, and BDNF. After 7, 10, or 14 days of mitogen withdrawal in the absence (control) or presence of 6.25 μg/ml OPN, NSC were fixed, then immunocytochemically stained for markers for neuron specific beta-III tubulin (anti-TuJ1; mouse monoclonal, dilution 1:100, R&D Systems), mature neurons (anti-MAP2; mouse monoclonal, dilution 1:1000, Sigma-Aldrich, Munich, Germany), astrocytes (anti-GFAP; rabbit polyclonal, dilution 1:2500, Abcam), oligodendrocytes (anti-CNPase; mouse monoclonal, clone 11-5B, dilution 1:500, Millipore, Billerica, USA), and undifferentiated stem cells (SOX2; mouse monoclonal, dilution 1:1000, R&D Systems). Besides staining for those single antigens, double-immunostaining was performed for glial fibrillary acidic protein (GFAP) plus 2′,3′-cyclic nucleotide 3′-phosphodiesterase (CNPase), in order to differentiate between astrocytes and oligodendrocytes in the same field of view. For visualization, fluorescein-labeled anti-mouse IgG or anti-rabbit IgG were used; all cells were additionally counterstained with Hoechst 33342. For quantification of the different fates of NSC, cells were counted manually in representative fields of view, taking into account those images displaying at least one specifically stained cell. Mean values ± SEM were established among equally treated samples.

### Real-time quantitative PCR

All quantitative PCR experiments were performed in quadruplicate.

RNA from cells was isolated using the RNeasy Mini Kit (Qiagen, Hilden, Germany) according to the manufacturer’s protocol. Total RNA concentration and purity were evaluated photometrically. Total RNA was converted to cDNA by reverse transcription with the Quantitect reverse transcription kit (Qiagen). The primer used for Ki67 was obtained from Biolegio (Nijmegen, The Netherlands). The sequences of the primers were: A) forward, TCTTGGCACTCACAGTCCAG, and B) reverse, GCTGGAAGCAAGTGAAGTCC. The quantitative PCR reaction was carried out using 10 ng total RNA in 20 μl reaction Quantitect Reagents (Qiagen) according to the manufacturer’s recommendations. The samples were amplified and quantified on a Rotorgene 2000 (Corbett, Sydney, Australia) using the following thermal cycler conditions: activation, 95 °C, 10 minutes; cycling, 50 cycles; step1, 92 °C, 15 s; step 2, 52 °C, 15 s; step 3, 72 °C, 40 s. PCR product integrity was evaluated by melting point analysis and agarose gel electrophoresis. Each sample was normalized to RPL13a as reference gene, and Ki67 mRNA levels were normalized to endogenous RPL13a expression (ΔCT); normalized values were then expressed as 2-ΔCt. Mean values ± SEM were calculated for treated and untreated cells.

### In vivo experiments

All animal procedures were in accordance with the German Laws for Animal Protection and were approved by the local animal care committee (Tierschutz-Ausschuss FZ Juelich) and local governmental authorities (LANUV NRW, AZ 84–02.04.2011.A169).

#### Photothrombosis

Adult male Fisher 344 rats weighing 200–300 g (n = 15) were subjected to photothrombosis as a model of focal cerebral ischemia. Animals were anesthetized by intraperitoneal injection of ketamine (75 mg/kg) and medetomidine (0.3 mg/kg), and placed in an atraumatic stereotactical frame. The scalp was incised to expose the skull surface. For illumination, a fiber-optic bundle with a 1.5 mm aperture was placed onto the skull, using the stereotactic coordinates 2 mm posterior and 3 mm lateral from bregma. The skull was illuminated with a cold, white light beam (150 W) for 20 minutes. During the first 2 minutes of illumination, the dye rose bengal (1 %, 1 μl/g bodyweight) was injected into the tail vein. Afterwards, the scalp incision was sutured, and animals were given an analgesic medication and allowed to recover from anesthesia. Then they were put back into their home cages, where they were given access to food and water ad libitum.

#### OPN treatment

To assess the effects of OPN on endogenous NSC, in vivo animals subjected to photothrombotic stroke and naïve adult male Fisher 344 rats weighing 200–300 g were treated with a single intracerebroventricular (i.c.v.) injection of either 500 μg recombinant OPN (R&D Systems) in 5 μl saline (stroke, n = 7; naïve, n = 5), or 5 μl saline only as control (stroke, n = 8; naïve, n = 5), using the stereotactic coordinates from bregma: antero-posterior −0.9 mm, medio-lateral +1.4 mm, ventro-dorsal +3.8 mm. In stroke rats, i.c.v. injection was performed in the same session as induction of photothrombosis. After surgery, animals were given an analgesic medication, were allowed to recover from anesthesia and were put back into their home cages, where they were given access to food and water ad libitum.

To label dividing (stem) cells in vivo, the tracer bromodeoxyuridine (BrdU) was injected subcutaneously every other day for 7 days at a dose of 50 mg/kg per injection, starting on the day after i.c.v. injection of OPN or placebo, respectively, leading to a cumulative dose of 200 mg/kg BrdU per animal.

Eight days after stroke and/or i.c.v. injection, rats were sacrificed under deep anesthesia. Brains were extracted and stored at −80 °C for further processing.

#### Immunohistochemistry

Coronal brain sections of 20 μm were cut throughout the entire brain and fixed with 4 % PFA. To quantify the number of endogenous NSC that had divided in the week following OPN treatment, sections were stained for BrdU incorporated into those cells (mouse monoclonal, clone BU-33, dilution 1:200, cat-# B2531, Sigma-Aldrich). For antigen-retrieval prior to BrdU-staining, sections were microwave-heated in 0.01 M citrate buffer, pH 6.0, for 5 minutes, followed by 2 N HCl at 37 °C for 30 minutes. To assess the effect of OPN on neurogenesis, sections were additionally stained for doublecortin (DCX; goat polyclonal, dilution 1:800, cat-# sc-8066, Santa Cruz Biotechnology, Dallas, USA). To assess the effect of OPN on synaptogenesis, the sections were stained for Synapsin-1 (rabbit polyclonal; dilution 1:1000, cat-#ab64581, Abcam). For visualization of all primary antibodies, the ABC Elite kit (Vector Laboratories, Burlingame, USA), with diaminobenzidine (Sigma-Aldrich) as the final reaction product, was used.

Representative images were taken throughout the SVZ using a digital microscope (Keyence BZ-9000E). The number of BrdU-positive cells in the SVZ was counted manually, and mean values ± SEM were established across animals of the same treatment group. To quantify neuroblasts in the SVZ, the area covered by DCX-positive cells was measured in μm^2^ using the software BZ-II Analyzer (Keyence).

### Statistical analysis

Descriptive statistics and Student’s t-tests were performed with Microsoft Excel 2003 (Microsoft Corp., Redmond, WA, USA). For comparison of multiple groups, one way analysis of variance (ANOVA) was performed, following Dunnett’s method for multiple comparisons. Statistical significance was set at the less than 5 % level (*p* < 0.05).

## Results

### Undifferentiated NSC abundantly expressed CXCR4

**Fig. 1:**
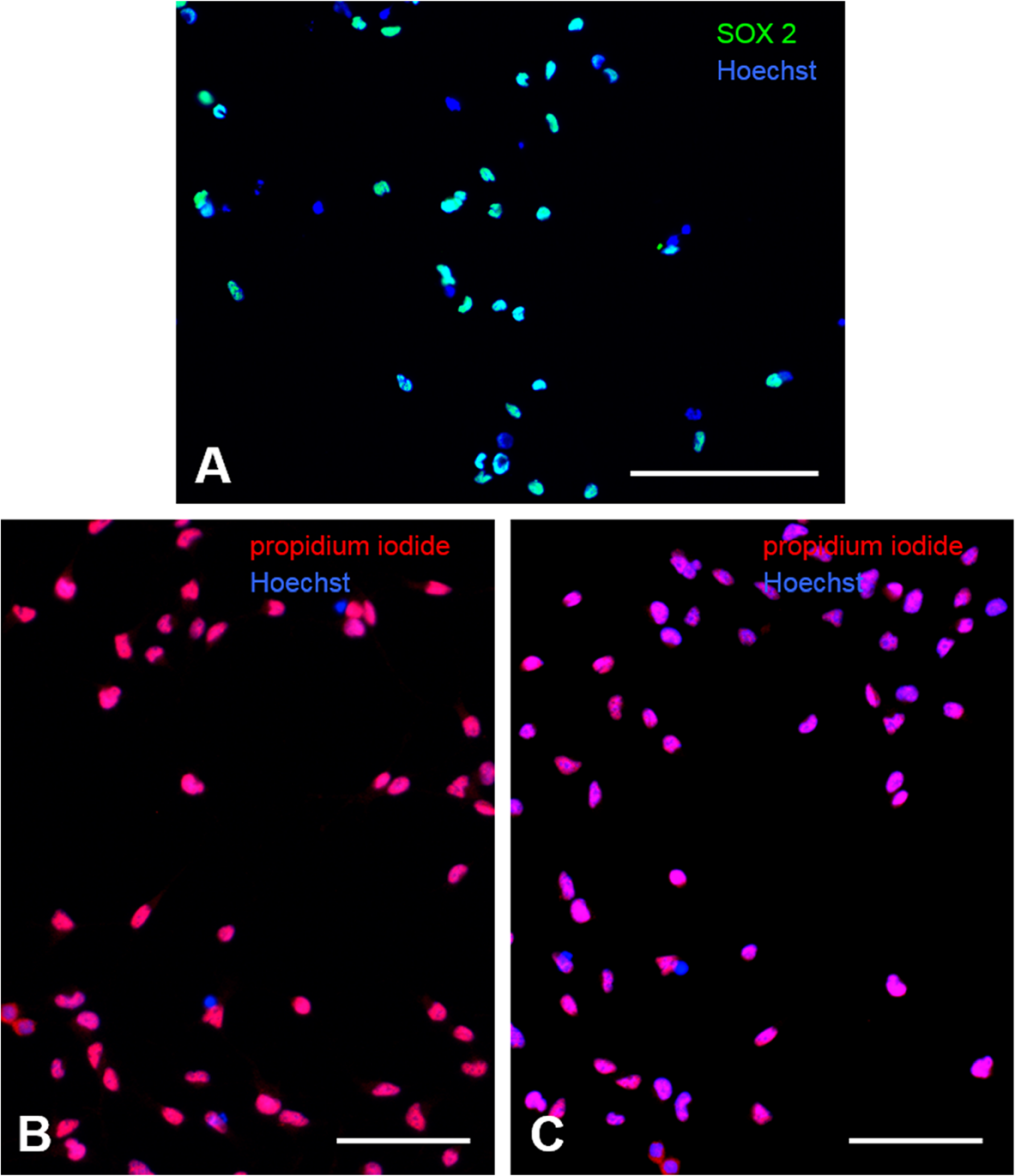
Undifferentiated NSC abundantly expressed CXCR4. **a** Primary fetal rat NSC expressed SOX2 as a marker for undifferentiated cells (green); cells were counterstained with Hoechst (blue; scale bar represents 100 μm). **b** Staining for CXCR4 (red) revealed that the cytokine receptor was expressed abundantly on primary NSC; cells were counterstained with Hoechst (blue; scale bar represents 50 μm). **c** Treatment with 6.25 μg OPN for 24 h did not change the abundant expression of CXCR4 (red) on NSC (blue: counterstaining with Hoechst; scale bar represents 50 μm)

Primary fetal rat NSC were grown in monolayer cultures and characteristically expressed SOX2 as a marker for undifferentiated cells (Fig. 1a). Immunocytochemical staining revealed that the cytokine receptor CXCR4 was expressed on nearly all primary NSC (Fig. 1b). This abundant expression was unaffected by treatment with 6.25 μg OPN for 24 h (Fig. 1c).

### OPN increased NSC numbers via CXCR4 signaling

**Fig. 2:**
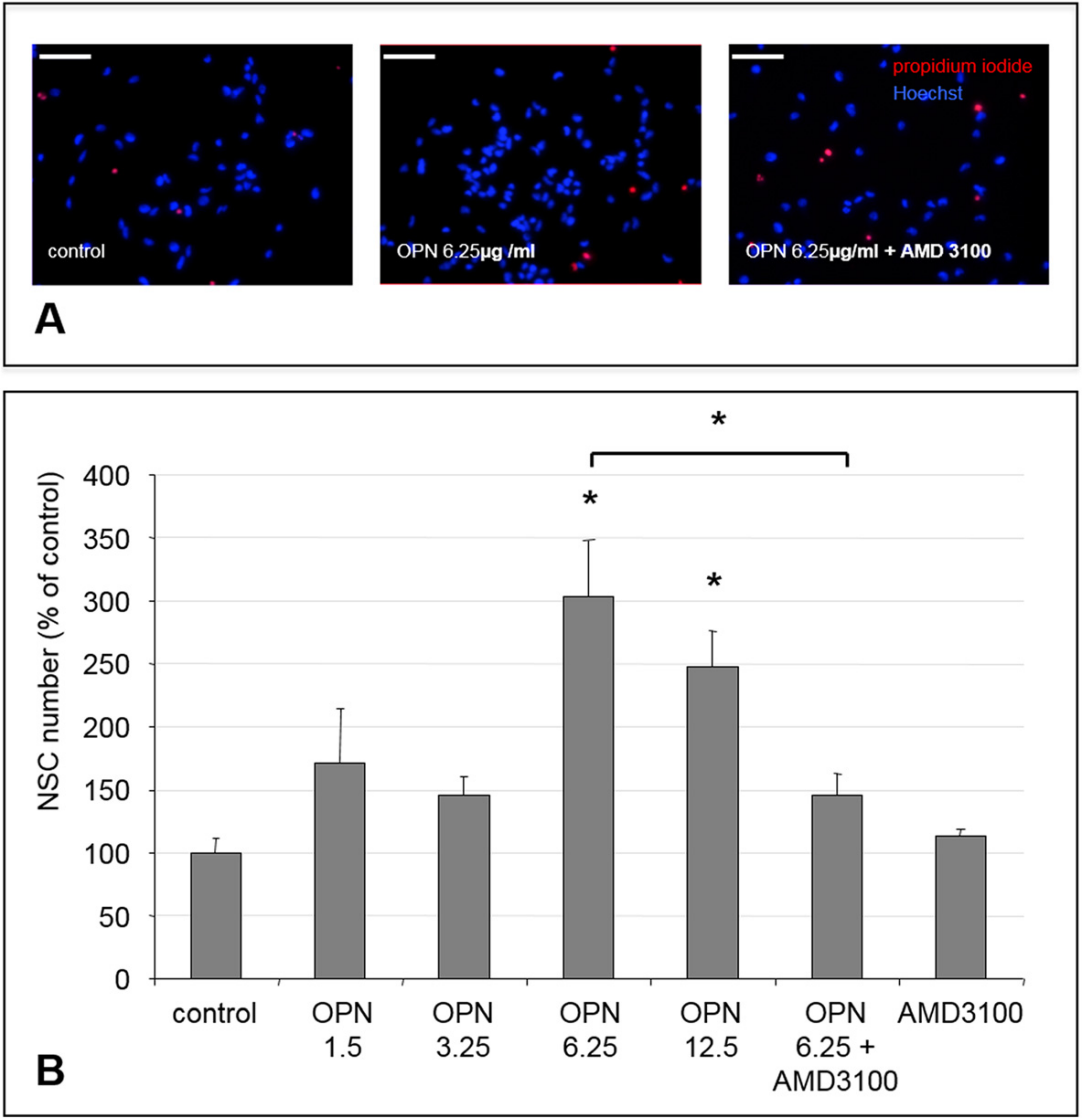
Osteopontin (*OPN*) increased neural stem cell (*NSC*) numbers via CXCR4 signaling. **a** *Left panel*: Untreated NSC (control) were stained with Hoechst (blue), while dead NSC were additionally labeled with propidium iodide (red), allowing calculation of a ratio of viable NSC. *Middle panel*: After treatment with 6.25 μg/ml OPN for 72 h, the number of viable NSC was increased. *Right panel*: Addition of AMD3100 to OPN-treated NSC abrogated this effect (scale bar represents 100 μm). **b** OPN at concentrations of 6.25 and 12.5 μg/ml significantly increased the numbers of NSC compared to control. Blocking CXCR4 with AMD 3100 for 1 h prior to OPN treatment completely abrogated the positive effect of OPN on NSC numbers, while AMD 3100 alone had no effect (values displayed as means ± SEM; *p < 0.05)

To investigate the effect of OPN on NSC numbers, cells were treated with OPN of various concentrations for 72 h. The number of viable NSC after this time was determined by co-staining cell nuclei with propidium iodide, thereby identifying and excluding dead cells (Fig. 2a). The number of viable NSC was significantly increased after treatment with 6.25 μg/ml and 12.5 μg/ml OPN (*p* < 0.05; Fig. 2a, b). Blockage of CXCR4 with 10 μM AMD 3100 1 h prior to OPN-treatment completely blocked the positive effect of OPN on NSC numbers (*p* < 0.05), while treatment with AMD 3100 alone (without OPN) had no impact on NSC numbers (Fig. 2a, b).

Since OPN at a concentration of 6.25 μg/ml led to the highest increase in cell number, we used this concentration for all further experiments.

### OPN increased both proliferation and survival of NSC

We further analyzed whether the positive effect of OPN on NSC numbers was due to increased cell proliferation, enhanced cell survival, or both.

**Fig. 3:**
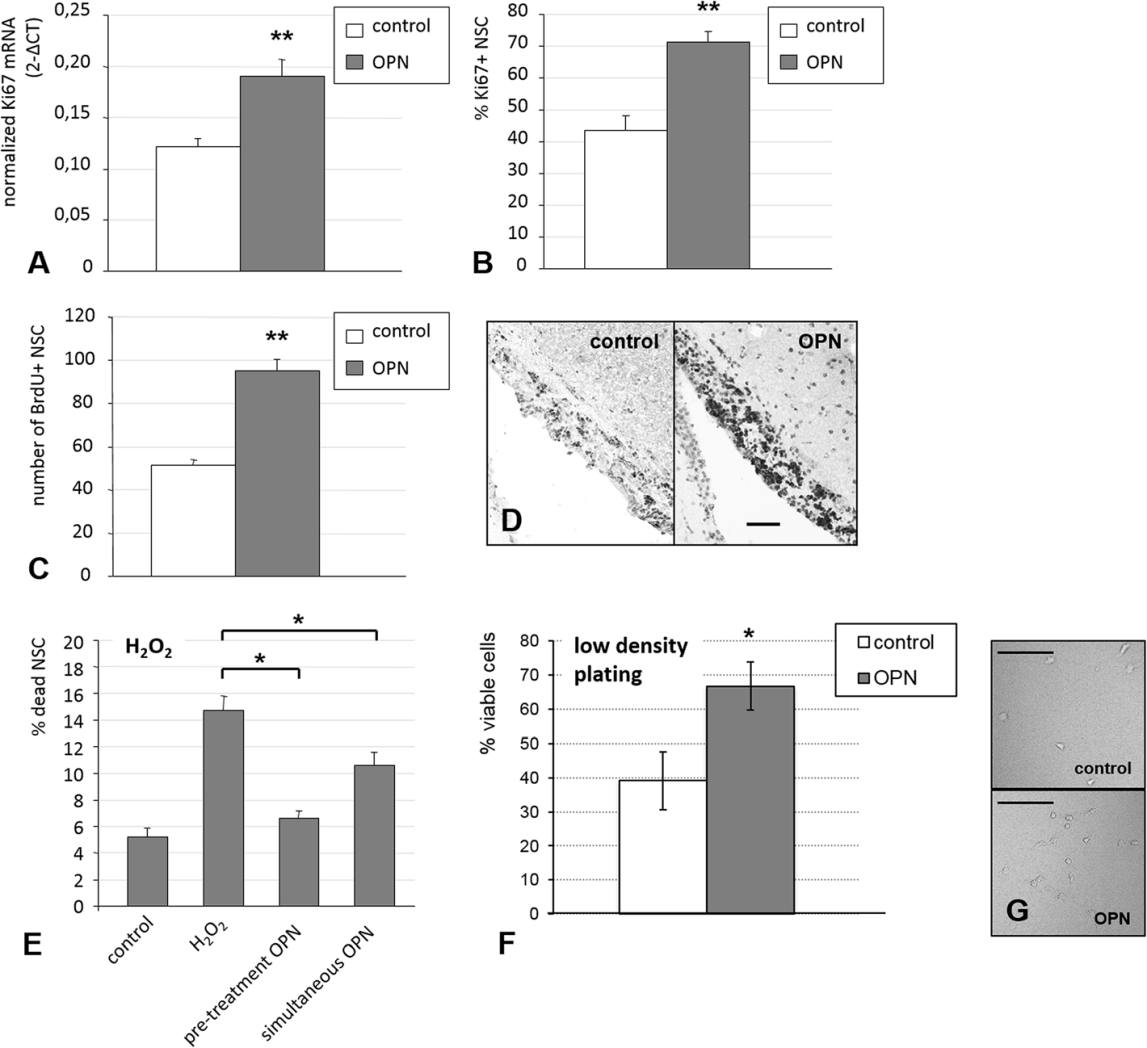
Osteopontin (*OPN*) increased both survival and proliferation of neural stem cells (*NSC*). **a** NSC were treated with OPN at 6.25 μg/ml for 72 h. Quantitative PCR revealed that OPN treatment led to an increase in Ki67-mRNA, compared to untreated NSC (values displayed as means ± SEM; **p < 0.01). **b** At the protein level, the percentage of Ki67-positive cells was significantly increased after treatment with 6.25 μg/ml OPN for 72 h (values displayed as means ± SEM; **p < 0.01). **c** Adult male rats injected with a single dose of 500 μg OPN intracerebroventricularly displayed a significantly higher number of proliferating NSC in the SVZ as the major NSC niche, corroborating the effects of OPN on NSC proliferation in vivo (values displayed as means ± SEM; **p < 0.01). **d** Representative images from the SVZ of rats treated with either placebo (*left*) or OPN (*right*; scale bar represents 200 μm). **e** NSC cultures were exposed to oxidative stress by H_2_O_2_ (300 nM for 24 h), increasing cell death as assessed by propidium iodide staining. Pre-treatment of NSC with 6.25 μg/ml OPN 24 h prior to oxidative stress completely rescued NSC from this toxicity, while simultaneously addition of OPN and H_2_O_2_ prevented about half the cells from dying (values displayed as means ± SEM; *p < 0.05). **f** NSC cultures were plated at very low density (266 cells/cm^2^). The numbers of living and dead cells were assessed by Hoechst and propidium iodide staining. Treatment with 6.25 μg/ml OPN led to a significantly higher number of viable NSC after 8 days of incubation (values displayed as means ± SEM; *p < 0.05). **g** NSC plated at very low density in the presence or absence of OPN at 6.25 μg/ml were assessed morphologically after 8 days. NSC adapted a slightly branched morphology under the stress conditions of very low density plating, independent of OPN treatment (scale bars represent 50 μm). *BrdU* bromodeoxyuridine

To study the effects of OPN on NSC proliferation, we analyzed the expression of the established proliferation marker Ki67 both on the mRNA and the protein level. Ki67-mRNA was quantitatively assessed after OPN treatment using real-time quantitative PCR. NSC treated with OPN at 6.25 μg/ml for 72 h yielded significantly more Ki67-mRNA than untreated NSC (*p* < 0.01; Fig. 3a). Immunocytochemically, the percentage of Ki67-positive cells was significantly increased after treatment with 6.25 μg/ml OPN for 72 h as compared to untreated cells, corroborating quantitative PCR data at the protein level (Fig. 3b).

To assess whether OPN exerted a similar effect on NSC proliferation in vivo, adult male Fisher rats were injected with a single dose of 500 μg OPN into the lateral ventricle of the brain, and compared to sham-injected animals. For the following 8 days, BrdU was repetitively administered systemically to label proliferating (stem) cells in vivo. Treatment with OPN led to a significant increase in BrdU-positive cells in the SVZ as the major NSC niche, indicating that the effects of OPN on NSC proliferation in vitro were reproducible in vivo (*p* < 0.01, Fig. 3c, d).

To assess a potential additional effect of OPN on the survival of NSC, we exposed NSC cultures to severe oxidative stress by adding 300 nM H_2_O_2_ for 24 h. This exposure led to an approximately threefold increase in the percentage of dead NSC, as assessed by propidium iodide staining (Fig. 3e). Pre-treatment of NSC with 6.25 μg/ml OPN 24 h prior to oxidative stress completely rescued the viability of NSC (*p* < 0.05; Fig. 3e). Even adding OPN simultaneously with H_2_O_2_ prevented about half of the cells from dying, confirming a positive effect of OPN on NSC survival during cell stress (*p* < 0.01; Fig. 3e). To confirm the positive effect of OPN on the survival of NSC under stress conditions, we additionally plated NSC at very low density and grew them in the presence or absence of OPN for 8 days. Treatment with 6.25 μg/ml OPN led to a significantly higher number of viable NSC (*p* < 0.05; Fig. 3f). Of note, NSC adapted a slightly branched morphology under stress conditions, which was unaffected by OPN (Fig. 3g).

### OPN promoted NSC migration via CXCR4 signaling

**Fig. 4:**
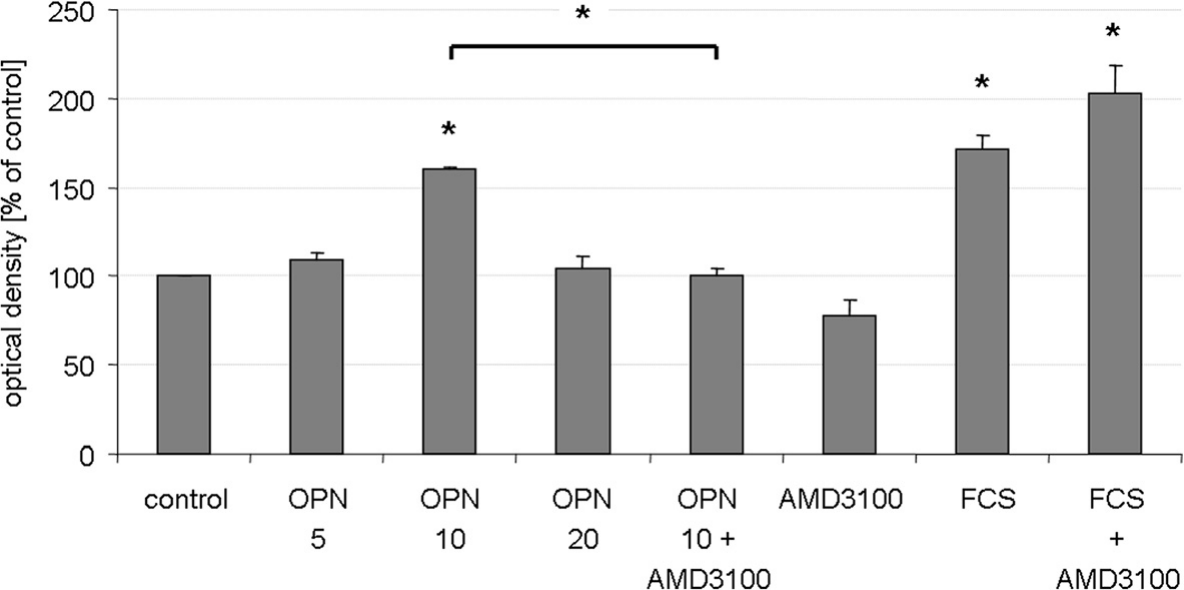
Osteopontin (*OPN*) promoted neural stem cell (*NSC*) migration via CXCR4 signaling. A modified Boyden chamber trans-well migration assay was used to assess the effects of OPN on NSC migration, measuring the optical density of migrating cells, corresponding to their number. Growing NSC in the presence of 10 μg/ml OPN for 48 h significantly increased their migration, while concentrations of 5 and 20 μg/ml did not have an effect. The positive effect of OPN on NSC migration was completely blocked by treatment with AMD 3100, indicating that the effect was mediated through CXCR4 receptor signaling. Fetal calf serum (*FCS*) served as positive control; its migration-promoting effect was unaffected by CXCR4 blockage. AMD 3100 alone did not affect NSC migration (values displayed as means ± SEM; *p < 0.05)

To assess the chemoattractant effect of OPN on NSC, a modified Boyden chamber trans-well migration assay was performed. NSC were treated with OPN at 5, 10 or 20 μg/ml for 48 h and compared to untreated cells as a negative control; FCS as an established potent chemoattractant was used as a positive control. Upon addition of OPN at a concentration of 10 μg/ml, a significantly higher number of NSC migrated to the lower chamber (*p* < 0.05, Fig. 4). This effect was completely abolished by blocking CXCR4 with AMD 3100 (*p* < 0.05), while the migration-promoting effect of FCS was unaffected by AMD 3100, suggesting specific signaling of OPN via CXCR4 (Fig. 4). AMD 3100 alone had no effect on NSC migration (Fig. 4).

### OPN promoted neurogenesis

**Fig. 5:**
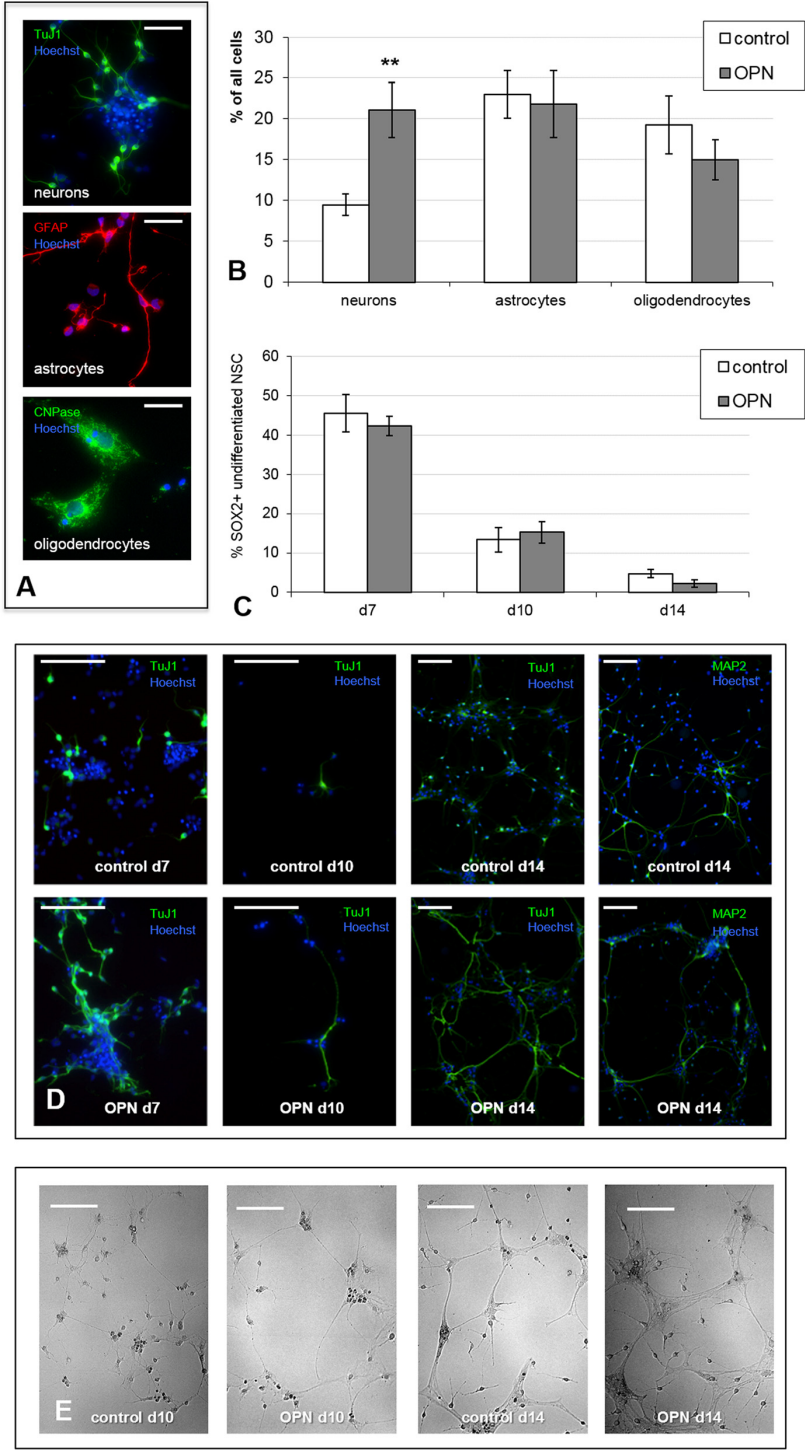
Osteopontin (*OPN*) promoted neurogenesis in vitro. **a** Neural stem cells (*NSC*) were allowed to differentiate for 7 days (*d*) after mitogen withdrawal, yielding all three cell fates: neurons (*above*, green), astrocytes (*middle*, red), and oligodendrocytes (*below*, green; scale bars represents 50 μm). **b** Constant exposure of NSC cultures to OPN at 6.25 μg/ml during differentiation led to increased neurogenesis, while other cell fates were slightly reduced, correspondently, albeit to a non-significant extent (values displayed as means ± SEM; **p < 0.01). **c** NSC were allowed to differentiate for 7, 10, and 14 days after mitogen withdrawal in the presence or absence of OPN at 6.25 μg/ml; the number of undifferentiated cells was subsequently assessed by staining against SRY (sex determining region Y) box 2 (*SOX2*). During that period, the number of undifferentiated cells continuously declined, regardless of the presence of OPN (values displayed as means ± SEM). **d** Generation of TuJ1-positive neurons (green) during differentiation was increased by OPN treatment (*lower row*) as compared to control (*upper row*) at days 7, 10, and 14 after mitogen withdrawal. During that period, the axon length grew notably and neurons began to form networks; both observations were more pronounced in OPN-treated cells. By day 14, mature MAP2^+^-positive neurons had formed (*right column*; scale bars represent 100 μm). **e** NSC were allowed to differentiate 10 and 14 days after mitogen withdrawal in the presence or absence of OPN at 6.25 μg/ml, and then their morphology was analyzed. Between day 10 and day 14 after mitogen withdrawal, NSC differentiation further proceeded, the neurons formed long processes and network-like formations. This effect was particularly pronounced in the OPN-treated cells (scale bars represent 100 μm)

To examine the effects of OPN on the differentiation potential of NSC, cells were treated with 6.25 μg/ml OPN, while untreated cells served as control. During the expansion phase, mitogen was withdrawn from NSC cultures to induce differentiation, and cell fate was determined immunocytochemically after 7, 10, and 14 days (Fig. 5a). Compared to untreated cells, the number of neurons doubled after OPN treatment (*p* < 0.01, Fig. 5b, d). In contrast, the other cell fates (i.e., astrocytes, oligodendrocytes) as well as the remaining undifferentiated cells were slightly reduced, albeit to a non-significant extent (Fig. 5b, c).

We further focused on the effect of OPN on neurogenesis at the late time points. From day 10 of differentiation onward, an escalating formation of long neuronal processes and network-like formations was observed. Notably, this development was enhanced by OPN treatment (Fig. 5d, e). Furthermore, after 14 days of mitogen withdrawal, several NSC had differentiated into mature neurons, as indicated by immunolabeling with MAP2. OPN-treated cells generated more neurons, which, in turn, displayed denser neuronal networks (Fig. 5b, d, e).

**Fig. 6:**
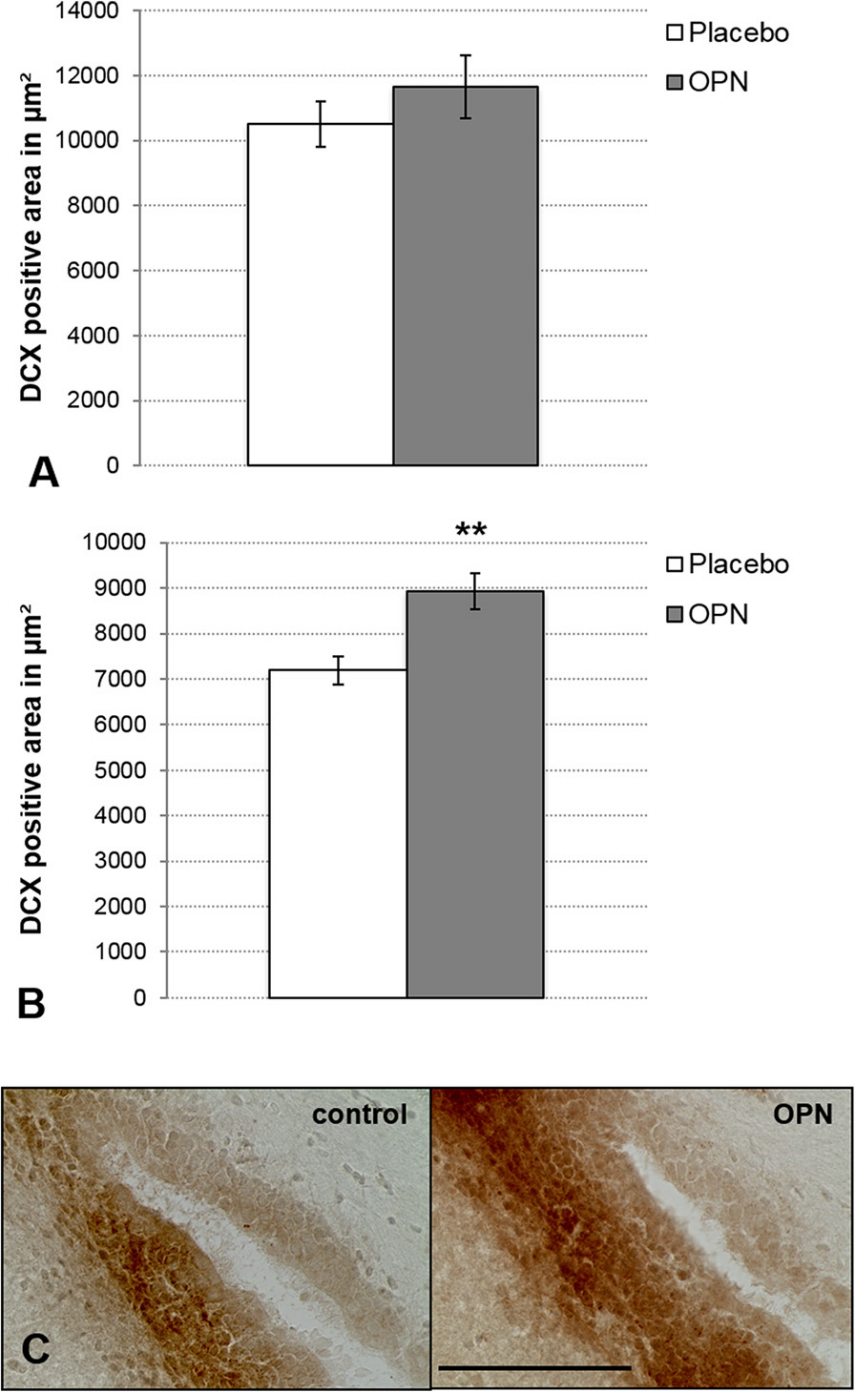
Osteopontin (*OPN*) promoted neurogenesis after stroke in vivo. **a** After single i.c.v. injection of 500 μg OPN in adult, unlesioned rats, OPN failed to significantly increase the area covered by doublecortin (*DCX*)-positive young neuroblasts in the SVZ. **b** In adults rats that underwent photothrombosis, a single i.c.v. injection of 500 μg OPN significantly increased the area covered by DCX-positive neuroblasts in the SVZ (values are displayed as means ± SEM, **p < 0.01). **c** Representative, DCX-stained images from the SVZ of rats subjected to cerebral ischemia, treated with either placebo (*left*) or OPN (*right*). OPN treatment led to an increase of neuroblasts in the SVZ (scale bar represents 100 μm)

To address the effect of OPN on neurogenesis in the rat brain in vivo, DCX-positive young neuroblasts in the SVZ were quantified 8 days after a single injection of 500 μg OPN into the lateral ventricle of the brain, while placebo-injected animals served as control. While OPN-treated animals displayed a slightly larger DCX-positive area of the SVZ compared to the placebo-treated controls, this effect was not statistically significant (Fig. 6a). Under the hypothesis that OPN might promote neurogenesis to a larger extent in a model of stem cell-mediated regeneration, we evaluated the effect of OPN on neurogenesis in a rat model of focal cerebral ischemia. When rats subjected to photothrombotic stroke received a single injection of 500 μg OPN into the lateral ventricle of the brain, OPN-treated animals displayed significantly more DCX-positive neuroblasts in the SVZ compared to the placebo-treated controls (*p* < 0.01, Fig. 6b, c).

Furthermore, we assessed the effect of OPN on synaptogenesis in the naïve rat brain in vivo. However, after 8 days, the synapsin-1-positive cells in the SVZ of OPN-treated naïve rats did not differ from control animals (Additional file 1: Figure S1).

## Discussion

We found OPN to enhance both survival as well as proliferation of NSC. Data suggest that OPN increases the numbers of NSC via the chemokine receptor CXCR4. OPN has previously been shown to promote survival of various cells including bone marrow cells [42], dendritic cells [43], and cancer cells [44]. As a mechanism of action, its binding to splice variants of the cell surface glycoprotein CD44, activating the downstream PI3K pathway, has been suggested [44, 45]. Our data extend these findings by showing that beyond those types of cells, the survival of NSC exposed to oxidative stress is increased by OPN as well. This pro-survival effect of OPN on NSC under oxidative stress might facilitate survival of these cells under ischemic conditions such as stroke. Consistent with this assumption, OPN has been reported to protect neurons from ischemic injury [18, 19].

Previous reports have discovered diverse effects of OPN on cell proliferation. On the one hand, OPN was shown to directly increase proliferation of various tumor cells [45, 46]. Likewise, absence of OPN has been linked to decreased cell proliferation of both tumor cells [47] and erythroblasts [48]. On the other hand, OPN has been shown to suppress proliferation of hematopoietic stem cells through the induction of quiescence [35]. Regarding NSC, thus far non-conclusive effects of OPN on proliferation have been reported: while some reports failed to detect any effect of OPN on NSC proliferation in vitro [41] or in vivo after experimental stroke [49], others showed that NSC proliferation was reduced in the absence of OPN in a mouse model of hypoxic brain injury [50], or that OPN directly induced proliferation of NSC grown as neurosphere cultures [39]. Kalluri and Dempsey suggested that these seemingly contradictory results could result from different experimental conditions: they demonstrated that OPN increased NSC proliferation in neurosphere cultures only in the presence of FGF2 [39]. In our study, we found OPN increased the proliferation in monolayer cultures of primary NSC grown in the presence of FGF2, and additionally in the rat brain after stroke in vivo. Moreover, we here report for the first time that these effects are mediated via the cytokine receptor CXCR4 present on NSC.

OPN is a potent chemoattractant for various types of stem cells such as mesenchymal [34] and hematopoietic stem cells [35]. Yan et al. found that in the absence of OPN less neuroblasts migrate towards an ischemic [40] or hemorrhagic brain lesion [41]. In cultured neurospheres, they found that blocking the integrin-β1 receptor inhibited OPN-induced migration [40], in line with a later report on OPN promoting the migration of mesenchymal stem cells via integrin-β1 [51]. In hepatocellular carcinoma, on the other hand, OPN was shown to promote cell migration through the CXCR4 receptor [38]. We here demonstrate that migration of primary NSC grown in homogenous monolayer cultures is also mediated through CXCR4. The effects of both CXCR4 and integrin-α4β1 on NSC migration are consistent with their known support of the migration of hematopoietic stem cells [52]. Relevant cross-talk between these two pathways has also been described for neutrophils [53] and tumor cells [54, 55], and seems to also occur in the brain during autoimmune disorders [56].

To our knowledge, this is the first report describing a direct pro-neurogenic effect of OPN on NSC. Yan et al. have previously described that neutralizing OPN led to a decreased number of neuroblasts in the ischemic striatum, attributing this effect to a decreased migration of neuroblasts from the SVZ towards the striatum [40]. Plantman et al. reported a positive effect of OPN on neurite outgrowth of mature hippocampal neurons [57]. Our described direct positive effect of OPN on neurogenesis constitutes a novel aspect in stem cell-mediated regeneration after stroke, since the inflammatory milieu elicited after focal cerebral ischemia has primarily been associated with a gliogenic fate of NSC [58, 59]. Up-regulation of OPN in the inflammatory environment after cerebral ischemia, as observed 3–6 days after stroke [14–17], might thus counteract the glial differentiation of NSC attracted towards the ischemic lesion, thereby promoting regeneration and repair. Interestingly, we observed a significant effect of OPN on neurogenesis in vivo in animals that had undergone experimental stroke. These findings further underline the concept of a regulatory role of OPN under inflammatory conditions.

## Conclusion

We here demonstrate positive effects of OPN on survival, proliferation, migration and neuronal differentiation of NSC. Notably, OPN increased neurogenesis both in vitro as well as in vivo after cerebral ischemia. Since OPN is expressed in the subacute stages of focal cerebral ischemia, its impact on NSC might physiologically serve to facilitate regeneration and recovery. Our data strongly suggest that further studies investigating the in vivo use of OPN for enhancing the brain’s capacity for self-repair are warranted.

## Additional file

Additional file 1: Figure S1: OPN did not influence synaptogenesis in vivo. OPN failed to significantly increase the area covered by Synapsin-1-positive cells in the SVZ after single i.c.v. injection of 500 μg OPN in adult rats (scale bar represents 100 μm).

## Abbreviations

BrdU: bromodeoxyuridine
CNPase: 2′,3′-cyclic nucleotide 3′-phosphodiesterase
CXCR4: CXC chemokine receptor type 4
DCX: doublecortin
FCS: fetal calf serum
FGF: fibroblast growth factor
GFAP: glial fibrillary acidic protein
i.c.v: intracerebroventricular
Ig: immunoglobulin
NSC: neural stem cells
OPN: osteopontin
PFA: paraformaldehyde
SDF: stromal cell-derived factor
WEM: standard error of the mean
SOX-2: SRY (sex determining region Y) box 2
SVZ: subventricular zone
