# Supplementary figures and images for: Osteopontin mediates survival, proliferation and migration of neural stem cells through the chemokine receptor CXCR4
Source: Stem Cell Res Ther. 2015 May 22;6(1):99. doi: 10.1186/s13287-015-0098-x (PMC4464234; doi:10.1186/s13287-015-0098-x)

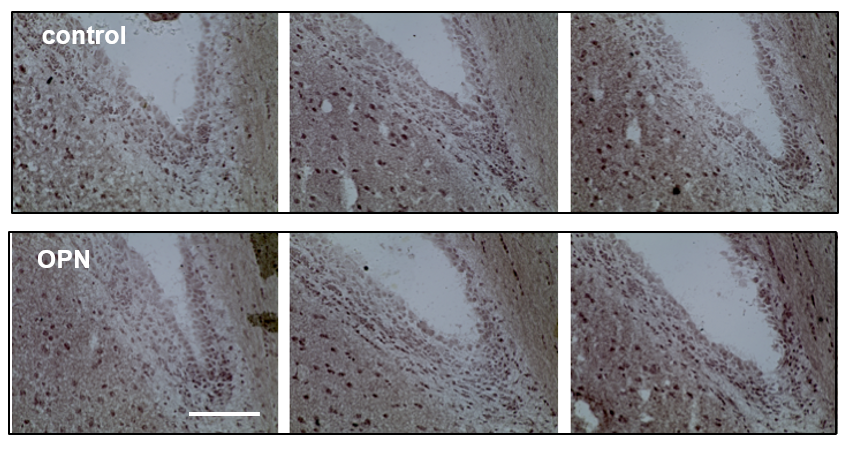

Supplement: Additional file 1: Figure S1: — OPN did not influence synaptogenesis in vivo. OPN failed to significantly increase the area covered by Synapsin-1-positive cells in the SVZ after single i.c.v. injection of 500 μg OPN in adult rats (scale bar represents 100 μm). [file 13287_2015_98_MOESM1_ESM.tiff]
